# Supplementary material for: Exploring TREC and KREC Levels in Nursing Home Residents and Staff and Their Association with SARS-CoV-2 Antibody Response After Vaccination
Source: Vaccines (Basel). 2025 Aug 19;13(8):874. doi: 10.3390/vaccines13080874 (PMC12390529; doi:10.3390/vaccines13080874)
Supplement: Supplementary file 1 [file vaccines-13-00874-s001.zip › vaccines-3765157-supplementary.pdf]

Supplementary File

Supplementary Table S1. Primers and probes sequences and concentrations and cycling conditions for the β-actin, T-cell Receptor Excision Circles (TREC) and Kappa-deleting Recombination Excision Circles (KREC) qPCR.

		Sequence (5'-3')	Concentration in Probe Mastermix (µM)	Cycling conditions
β-actin	Forward primer	GGA-TGC-AGA-AGG-AGA-TCA-CTG	0.5	- 30 s at 95°C<br>- 45 cycles:<br>30 s at 95°C<br>10 s at 56°C<br>30 s at 72°C.<br>- 5 min at 40°C
	Reverse primer	CGA-TCC-ACA-CGG-AGT-ACT-TG	0.5	
	Probe	ATTO 425 - CCC-TGG-CAC-CCA-GCA CAA-TG - BHQ®-1	0.4	
TREC	Forward primer	CAC-ATC-CCT-TTC-AAC-CAT-GCT	0.5	- 2 min at 50°C<br>- 10 min at 95°C<br>- 50 cycles:<br>15 s at 95°C<br>30 s at 62°C<br>30 s at 72°C<br>- 30 s at 40°C
	Reverse primer	GCC-AGC-TGC-AGG-GTT-TAG-G	0.5	
	Probe	6-FAM - ACA-CCT-CTG-GTT-TTT GTA-AAG-GTG-CCC-ACT - TAMRA	0.125	
KREC	Forward primer	GTG-AGG-GAC-ACG-CAG-CC	0.3	- 15 min at 95°C<br>- 50 cycles:<br>30 s at 95°C<br>30 s at 60°C<br>30 s at 72°C<br>- 30 s at 40°C
	Reverse primer	TCC-CTT-AGT-GGC-ATT-ATT-TGT-ATC-ACT	0.3	
	Probe	HEX-TCT-GCA-CGG-GCA-GCA-GGT-TGG-TAMRA	0.15	

Supplementary Table S2: Sequence of the synthetic DNA fragments used as standard sequence in qPCR.

	Sequence (5'-3')	Length (base pairs)

**β-actin**	GGATGCAGAAGGAGATCACTGCCCTGGCACCCAGCACAATGAAG ATCAAGGTGGGTGTCTTTCCTGCCTGAGCTGACCTGGGCAGGTCG GCTGTGGGGTCCTGTGGTGTGTGGGGAGCTGTCACATCCAGGGTC CTCACTGCCTGTCCCCTTCCCTCCTCAGATCATTGCTCCTCCTGAG CGCAAGTACTCCGTGTGGATCG	**202**
**TREC**	CACTATTGACTAGCTTGGAACTTCGCCACATCCCTTTCAACCATGCT GACACCTCTGGTTTTTGTAAAGGTGCCCACTCCTGTGCACGGTGAT GCATAGGCACCTGCACCCCGTGCCTAAACCCTGCAGCTGGC	**134**
**KREC**	CCAGTCACTTCGTGAGGGACACGCAGCCGTCTGCACGGGCAGCA GGTTGGCGTGCGCTGATGCATACATGACATGCAGTATTCACCTGCA CCCATGTGAGTGATACAAATAATGCCACTAAGGGA	**125**

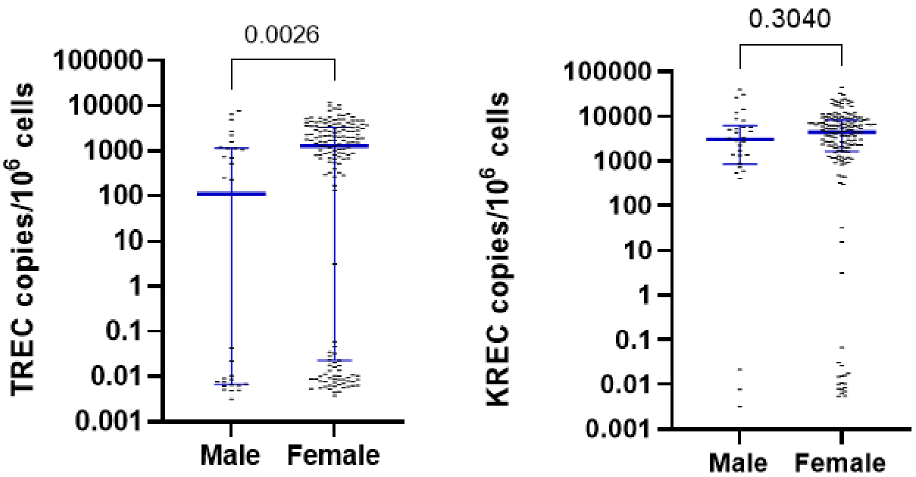

Supplementary Figure S1. T-cell Receptor Excision Circles (TREC) (left) and Kappa-deleting Recombination Excision Circles (KREC) (right) in males versus females. Blue horizontal lines represent the median value with interquartile range (IQR).

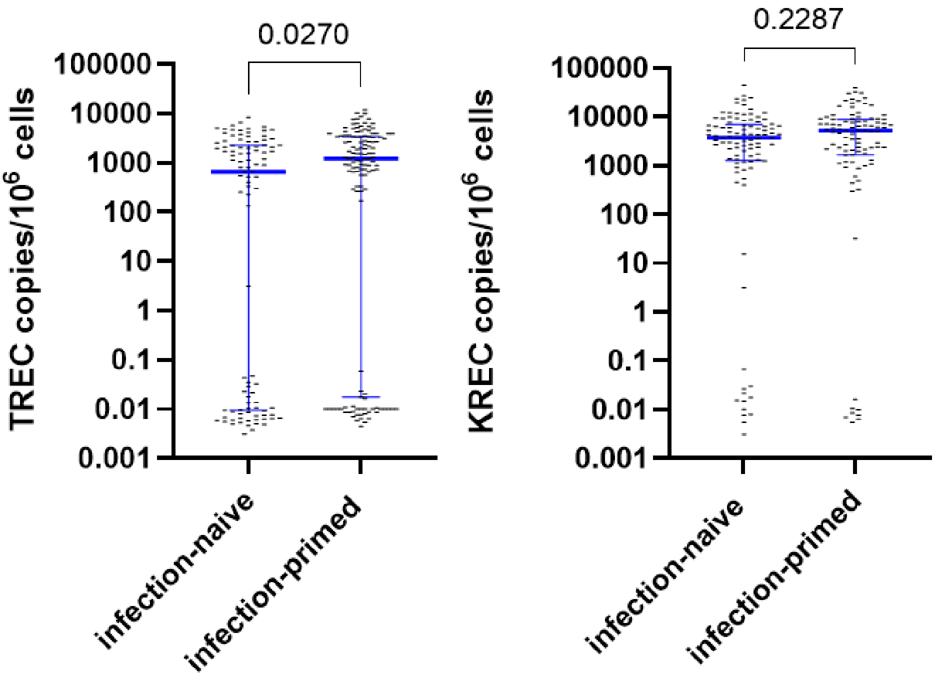

Supplementary Figure S2. T-cell Receptor Excision Circles (TREC) (left) and Kappa-deleting Recombination Excision Circles (KREC) (right) in infection-naïve and infection-primed subjects. Blue horizontal lines represent the median value with interquartile range (IQR).

Supplementary Table S3. Multiple linear regression models for anti-spike SARS-CoV-2 IgG antibodies (S1RBD IgG) with TREC (T-cell Receptor Excision Circles) and KREC (Kappa-deleting Recombination Excision Circles) as covariates in NHR (n=50) and NHS (n=148). Statistically significant p-values are underlined.

**Model 1 (TREC)**	Variable	Estimate	Standard error	95% Confidence Interval	P value
	Intercept	3,241	0,5774	2,102 to 4,381	<ins>≤0,0001</ins>
	Age (years)[a]	0,006573	0,006965	-0,007172 to 0,02032	0,3466
S1RBD IgG concentration[a]	TREC[b] (copies/10^6 cells)	-0,1630	0,3207	-0,7959 to 0,4699	0,6119
	Female	0,07066	0,1089	-0,1443 to 0,2856	0,5174
	Infection-primed	0,5459	0,07841	0,3912 to 0,7006	<ins>≤0,0001</ins>
**Model 2 (KREC)**					
	Intercept	3,163	0,1933	2,782 to 3,545	<ins>≤0,0001</ins>
	Age (years)[a]	-0,002652	0,002078	-0,006753 to 0,001450	0,2037
S1RBD IgG concentration[a]	KREC[b] (copies/10^6 cells)	0,0007618	0,001076	-0,001362 to 0,002886	0,4801
	Female	-0,07103	0,1106	-0,2893 to 0,1473	0,5217
	Infection-primed	0,5406	0,07926	0,3842 to 0,6970	<ins>≤0,0001</ins>

[a] Age and S1RBD were $\log_{10}$-transformed, [b] TREC and KREC levels underwent a Box-Cox transformation with lambda = 0.1 and lambda = 0.4, respectively.

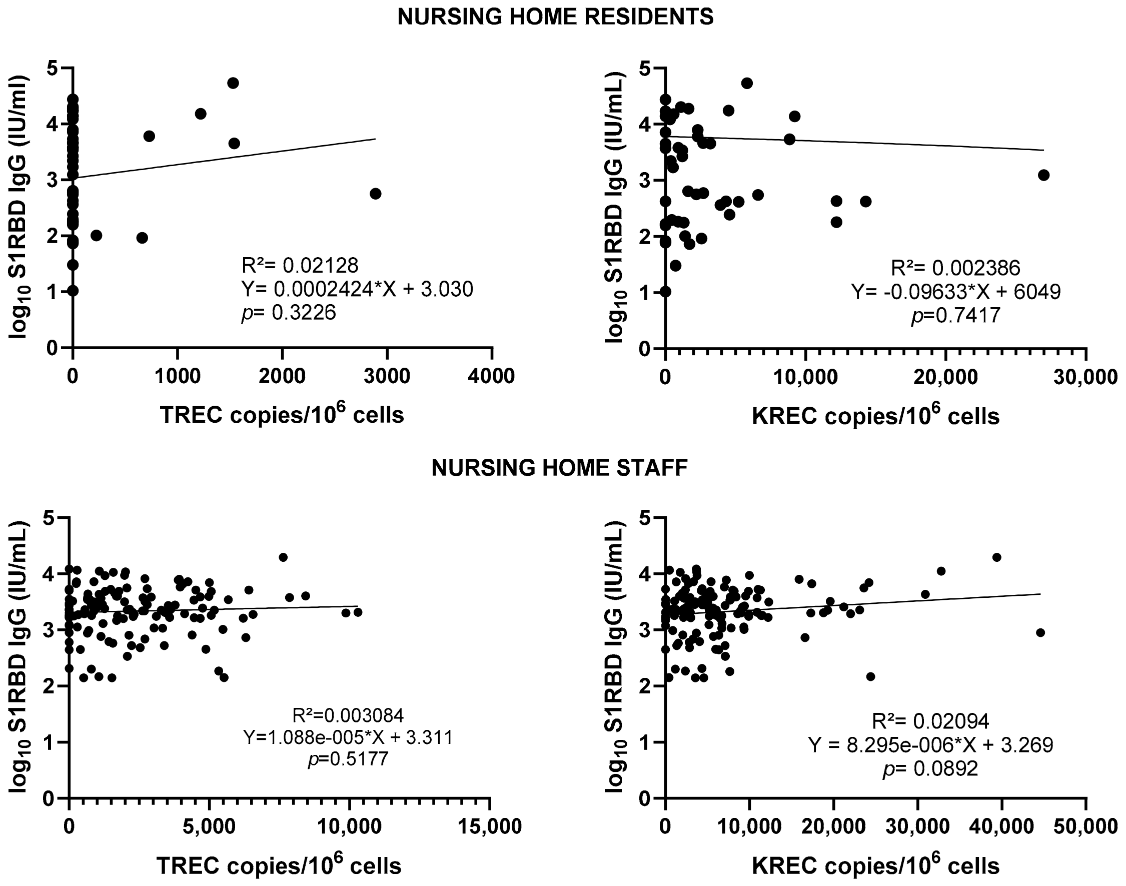

Figure S3. Simple linear regression between T-cell receptor excision circle (TREC) (left) and kappa-deleting recombination excision circle (KREC) (right) levels and S1RBD IgG antibody concentrations after COVID-19 vaccination separate for nursing home residents (n = 50) (above) and nursing home staff (n = 148) (below). International units/mL: IU/mL. The correlation coefficient (R^2), the equation representing the relationship between log10 S1RBD IgG levels (Y) and TREC or KREC levels (X), and *p*-value are shown in this figure. A *p*-value ≤0.05 is considered statistically significant.

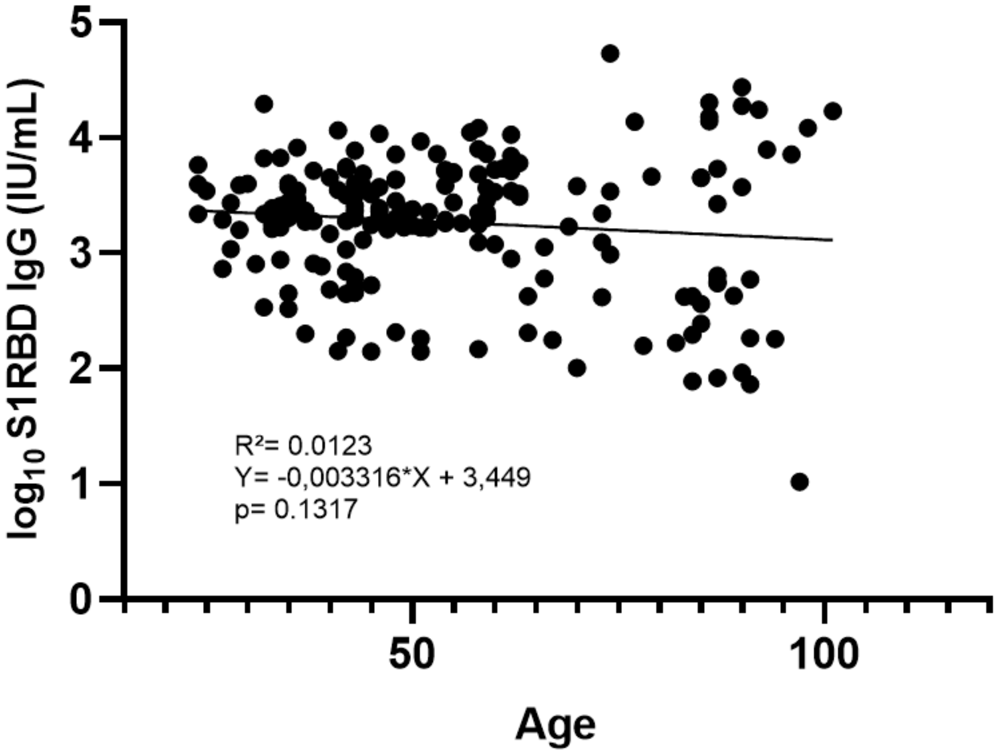

Supplementary Figure S4. Simple linear regression between age and anti-spike SARS-CoV-2 IgG antibodies (S1RBD IgG) in NHR (n=50) and NHS (n=148). The correlation coefficient (R^2), equation and p-value are shown in the figure.
